# Expression and clinical significance of miR-141-5p as a biomarker in the serum of patients with early spontaneous abortion

**DOI:** 10.1016/j.clinsp.2024.100327

**Published:** 2024-02-08

**Authors:** XiaoQun Che, Xiao Wang, LiLian Wang, LiHua Xu, Lin Zou, TianZhong Ma, Bi Chen

**Affiliations:** a Department of Reproductive Medicine, Affiliated Hospital of Guangdong Medical University, Zhanjiang City, Guangdong Province, China; b Department of Reproductive Medicine, Shunde Women and Children’s Hospital (Maternity and Child Healthcare Hospital of Shunde Foshan), Guangdong Medical University, Foshan, Guangdong Province, 528300, China

**Keywords:** Early spontaneous abortion, Progesterone, miR-141-5p, β-HCG, Estrogen

## Abstract

**Aim:**

miR-141-5p expression in patients with Early Spontaneous Abortion (ESA) and its correlation with hormone levels during pregnancy were investigated.

**Methods:**

A total of 70 pregnant women with ESA were selected as the research group, and 70 normal pregnant women who chose abortion for non-medical reasons were selected as the Con group. Serum β-HCG, Progesterone (P), and Estrogen (E2) were detected by enzyme-linked immunosorbent assay. Differentially expressed miRNAs were screened by miRNA microarray analysis. miR-141-5p expression was detected by RT-qPCR, and its correlation with serum β-HCG, P, and E2 levels was analyzed. The diagnostic value of miR-141-5p for ESA was evaluated by the ROC curve.

**Results:**

Serum β-HCG, P, and E2 were decreased and serum miR-141-5p was increased in patients with ESA. Pearson correlation analysis showed that serum β-HCG, P, and E2 levels were negatively correlated with miR-141-5p expression levels. ROC curve showed that miR-141-5p had a diagnostic value for ESA.

**Conclusions:**

miR-141-5p is related to hormone levels during pregnancy and is expected to become a new candidate diagnostic marker for ESA.

## Highlights

• Correlation analysis of miR-141-5p and serum β-HCG, P4 and E2 levels in patients with ESA.
• miR-141-5p as a diagnostic biomarker for ESA.
• β-HCG, P4 and E2 levels.

## Introduction

Early Spontaneous Abortion (ESA) occurs in the first trimester (before 12 weeks) and is considered a common complication during pregnancy.^1^ Statistics show that miscarriage constitutes a great proportion of all pregnancies, of which ESA accounts for more than 79 % of cases.^2^ Numerous studies have focused over the years on factors related to ESA, such as embryonic chromosomal,^3^ diabetes,^4^ endocrine, reproductive immune,^5^ infection,^6^ and maternal-fetal interface.^7^ Immune factors and endocrine hormone imbalance during pregnancy are important causes of early spontaneous abortion, accounting for 48 %‒59 % of them.^8^ Termination of pregnancy is the only viable treatment after diagnosis of spontaneous abortion by high-resolution dynamic transvaginal ultrasound.^9^^,^^10^ Therefore, the potential risk of ESA needs to be predicted early to improve the live birth rate of patients with ESA. Early diagnosis of ESA can improve the management of pregnant women, reduce the risk of miscarriage, prevent related complications, and carefully monitor ESA and pregnancy progression.^11^^,^^12^ Serum β‐Human Chorionic Gonadotropin (β‐HCG) has been widely used in early pregnancy assessment,^13^ However, it has been suggested that serum β-hCG is moderately predictive of ESA (AUC = 0.748, specificity = 71.13 %, sensitivity = 67.71 %).^14^ Therefore, there is an urgent need to find better diagnostic biomarkers associated with this pregnancy complication.

MicroRNAs (miRNAs) are small RNAs lacking coding potential that serve as key post-transcriptional regulators in a range of physiological and pathological settings,^15^ and they can regulate the expression of roughly 30 % of genes in various contexts.^16^ Importantly, miRNAs exhibit relatively high stability in biofluids and tissue samples, highlighting their potential utility as prognostic or diagnostic biomarkers in many diseases such as cancer,^17^ inflammation,^18^ and cardiovascular diseases,^19^ with certain miRNAs having further been identified as targets for preclinical therapeutic intervention.^20^ A considerable number of investigations suggest that abundant miRNA expression is detectable within the placental villus, decidua, and peripheral blood of humans, wherein these non-coding RNAs may contribute to SA pathogenesis by disrupting the function of extravillous trophoblasts.^21^, ^22^ highlighting their potential value as biomarkers of SA. In recent years, circulating miRNAs have been described as potential biomarkers in SA patients.^23^ For example, it has been studied that has-let-7c and has-miR-122 are up-regulated in maternal plasma, while has-miR-135a is down-regulated, which could be used as biomarkers in research for non-invasive prenatal diagnosis.^24^ miR-146b-5p and miR-520 h in the serum of women with recurrent SA are up-regulated.^25^ miR-141-5p is highly expressed in decidual NK cells of patients with recurrent spontaneous abortion, which may be related to recurrent spontaneous abortion.^26^ However, the clinical significance of miR-141-5p in ESA remains unclear.

Therefore, this study aims to explore miR-141-5p in ESA and its correlations with serum levels of β-HCG, P, and E2. In addition, the diagnostic accuracy of miR-141-5p as a biomarker for ESA was analyzed. These findings will provide new insights into ESA prevention and diagnosis.

## Methods

### Clinical sample collection

The research group included 50 pregnant women with ESA who were diagnosed at the Affiliated of Guangdong Medical University from February 1, 2016 to March 1, 2019. The ESA patients were 22‒40 years old, with an average age of 25.80 ± 7.48 years. The control group consisted of 50 normal pregnant women who underwent abortion induction during the same period. Normal pregnant women were 22‒38 years old, with an average age of 26.15 ± 5.32 years. Patients without miscarriage due to chromosome, anatomical structure, reproductive system infections, endocrine abnormalities, and autoimmune diseases were included, and those with HBV, AIDS, or infectious diseases were excluded. Patients with a history of abnormal pregnancy were excluded from the Con group. Subjects signed informed consent. This study was performed in accordance with the ethical standards in the *Declaration of Helsinki*, and the study protocol was approved by the ethics committee of Affiliated of Guangdong Medical University (Clinical trial Registration number: ChiCTR1900023427). In addition, this study is a clinical observational study, following the Strengthening the Reporting of Observational Studies in Epidemiology (STROBE) guidelines.

### Reagents and instruments

P, E2, and serum β-HCG were detected using ELISA kits (Qingdao Jieshikang Biotechnology Co., Ltd.) in combination with a microplate reader (Bio-Rad). miR-141-5p expression was determined using TRIzol (Nanjing Kebai Biotechnology, Nanjing, China), reverse transcription kit (Nanjing Kebai Biotechnology), miR-141-5p PCR kit (Thermo Fisher Scientific, MA, USA), and real-time PCR (Xi'an Tianlong Technology Co., Ltd., Xi'an, China).

### ELISA

Before 9:00 in the morning, fasting cubital venous blood was taken, centrifuged at 3010 × g at 4 °C for 5 min, and stored at 4 °C. The experimental procedures followed the protocol of ELISA kits. The OD value was immediately analyzed on the microplate reader at 450 nm.

### miRNA microarray analysis

Serum of patients in the Con group (*n* = 3) and the Stu group (*n* = 3) were obtained. Mircury Hy3/Hy5 Power labeling kit (Exiqon) was applied for labeling, and the Mircury LNA array chip (v. 18.0) was for hybridization. Images were obtained using a Genepix 4000B laser scanner and digitized using Genepix Pro 6.0 software (Axon Instruments). The heatmap is generated in Java Treeview.

### RT-qPCR

Total RNA was extracted from serum using the TRIzol method and reverse transcribed into cDNA using a reverse transcription kit. According to the instructions of miR-141-5p SYBR-Green PCR kit, miR-141-5p expression in the serum was detected. The reaction program was: 95 °C for 1 min, 95 °C for 15 s, and 60 °C for 20 s, in total of 39 cycles. miR-141-5p expression was normalized to U6 and calculated by the 2^−ΔΔct^ method. The primers are shown in Table 1.

**Table 1:** Primers.

Genes	Primers (5′–3′)
miR-141-5p	Forward: GCGCATCTTCCAGTACAGT
	Reverse: GCAGGGTCCGAGGTATTC
U6	Forward: CTCGCTTCGGCAGCACA
	Reverse: AACGCTTCACGAATTTGCGT

### Statistical analysis

Experimental data were assessed by SPSS 17.0 (Asia Analytics). The data are presented as the mean ± Standard Deviation (SD). Significant differences were calculated using Student's *t*-test. Correlations were analyzed by Pearson correlation. The diagnostic value was evaluated using the Receiver Operating Characteristic (ROC) curve; *p <* 0.05 indicated that the data difference was statistically significant.

## Results

### Patient pathological data

No significant differences were found in age, days of pregnancy, number of pregnancies, delivery, and abortion between the Stu group and Con group (Table 2).

**Table 2:** Pathological data of patients.

Characteristics	Control group (*n* = 50)	Study group (*n* = 50)	p
Age (years)	26.15 ± 5.32 (22‒38)	25.80 ± 7.48 (22‒40)	0.788
Days of pregnancy	47.5 ± 5.62 (41‒58)	49.2 ± 8.84 (40‒60)	0.254
Number of pregnancies	2.6 ± 1.60 (1‒5)	3.2 ± 1.80 (1‒6)	0.081
Number of deliveries	0.8 ± 0.65 (0‒2)	0.8 ± 0.72 (0‒2)	1
Number of abortions	0.9 ± 0.78 (0‒2)	1.2 ± 1.06 (0‒3)	0.11

### β-HCG, P and E2 levels

β-HCG, P, and E2 in the Stu group (4680.00 ± 1530.00 mU/L, 32.26 ± 11.45 nmoL/L, 95.84 ± 14.97 nmoL/L) were lower than those in the Con group (10,200.00 ± 3800.00 mU/L, 106.80 ± 42.30 nmoL/L, 288.36 ± 26.58 nmoL/L) (*p <* 0.001) (Table 3).

**Table 3:** Baseline characteristics and clinical parameters of subjects.

Factor	Control group (*n* = 50)	Study group (*n* = 50)	p
β-HCG (mU/L)	10,200 ± 3800	4680 ± 1530	<0.001
Progesterone (nmoL/L)	106.8 ± 42.3	32.26 ± 11.45	<0.001
Estrogen (ng/L)	288.36 ± 26.58	95.84 ± 14.97	<0.001

### Serum miR-141-5p in patients with ESA

**Fig. 1:**
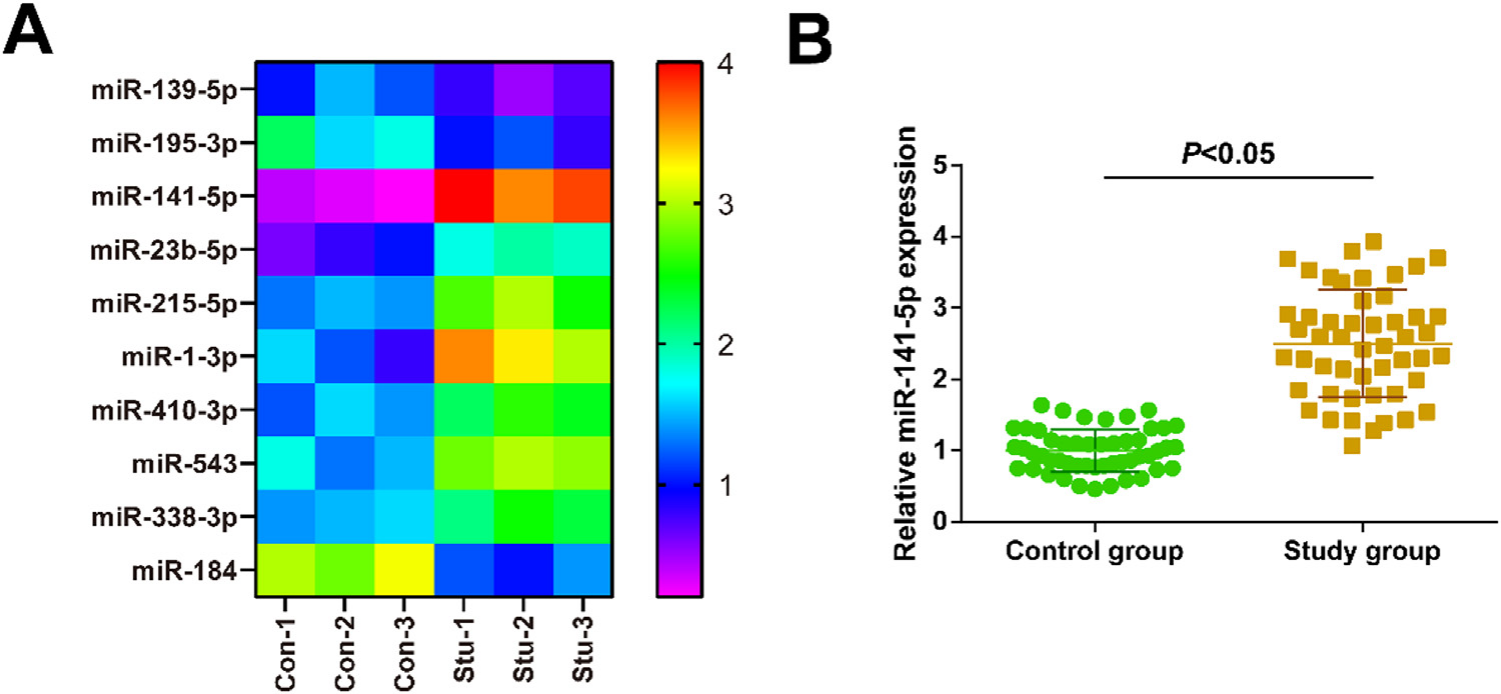
Serum miR-141-5p in patients with ESA (A: miRNA microarray analysis; B: RT-qPCR detection of miR-141-5p in the serum of patients with ESA). The data were all measurement data expressed as mean ± standard deviation). (For interpretation of the references to color in this figure legend, the reader is referred to the web version of this article.)

miRNA microarray analysis found differentially expressed miRNAs in the serum of ESA patients. Differential expression of miR-141-5p showed the most significance. RT-qPCR detected increased miR-141-5p in the serum of patients with ESA (Fig. 1A and B).

### Correlation analysis of miR-141-5p and serum β-HCG, P, and E2 levels in patients with ESA

**Fig. 2:**
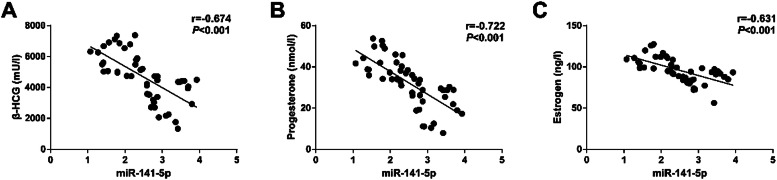
Correlation analysis (A‒C: Relation between miR-141-5p and serum β-HCG, P, and E2 levels in patients with ESA).

Pearson analysis found that serum miR-141-5p was negatively correlated with β-HCG levels (*r =* −0.674), P levels (*r* = −0.722), and E2 levels in ESA patients (*r =* −0.631) (*p <* 0.001; Fig. 2A‒C).

### miR-141-5p as a diagnostic biomarker for ESA

**Fig. 3:**
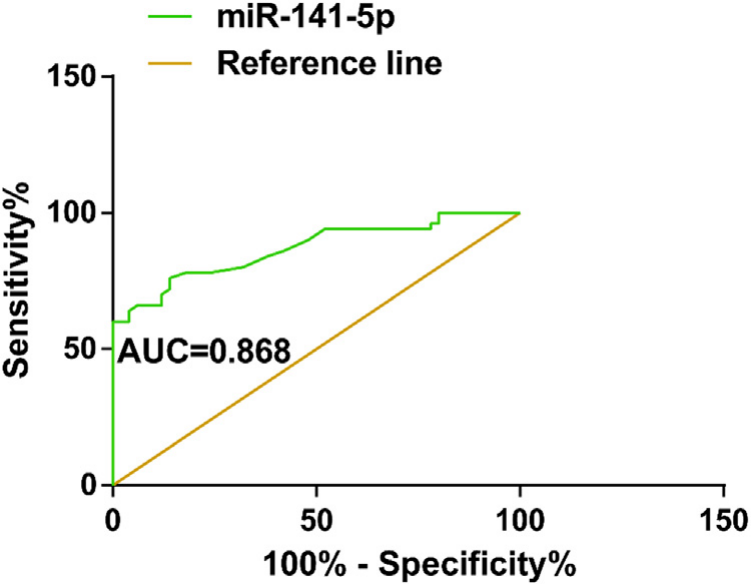
miR-141-5p as a diagnostic biomarker for ESA (ROC curve analysis to assess the diagnostic value of miR-141-5p for ESA). (For interpretation of the references to color in this figure legend, the reader is referred to the web version of this article.)

ROC analysis evaluated the diagnostic value of miR-141-5p, reporting that miR-141-5p had a diagnostic value for ESA (AUC = 0.868; specificity = 87.25 %, sensitivity = 82.60 %; SE = 0.036; 95 % CI = 0.797‒0.939; cutoff value = 1.545; *p <* 0.001) (Fig. 3).

## Discussion

Circulating miRNAs mediate epigenetic changes in human disease.^27^ There is potential for miRNAs to serve as biomarkers for detecting diseases associated with pregnancy.^28^ The value of miRNAs lies not only in their non-invasiveness but also in their stability and unchanged concentrations during pregnancy.^29^ As sequencing technology advances, more and more studies are looking at biomarkers that can be used to predict pregnancy-related diseases.^30^, ^31^ Therefore, this study attempted to find a specific biomarker for ESA diagnosis and treatment and to analyze its clinical significance. miR-141-5p, a member of the miR-200 family,^32^ has become increasingly evident in recent years that miR-141-5p plays a role in pregnancy-related disease. For example, miR-141-5p promotes the pathological process of preeclampsia^33^ and is involved in the regulation of EMT in endometriosis.^34^ miR-141-5p is possibly associated with P and P receptors in endometriosis.^35^ Furthermore, miR-141-5p can upregulate IL-36 in human primary trophoblast cells, which is involved in placental processes and responses to infection.^36^ Notably, in patients with recurrent SA, miR-141-5p is highly expressed in decidual NK cells.^26^ These findings suggest that miR-141-5p plays a role in fertility and reproduction.

This study first analyzed clinical data of patients in the Stu group and the Con group, finding no difference in age, days of pregnancy days, and numbers of pregnancies, delivery, and abortion. Studies have shown that pregnancy hormones such as β-HCG, P, and E2 are related to pregnancy maintenance.^37^ Currently, β-HCG, P, and E2 have been widely used for early pregnancy diagnosis.^13^, ^38^, ^39^ This study measured lower β-HCG, P, and E2 in ESA patients. Hormone levels in normal pregnancy are higher than those in patients with first-trimester miscarriage,^36^ which is consistent with the present findings.

To investigate whether miR-141-5p has diagnostic potential for ESA, this study performed miRNA microarray analysis on patients’ serum and found that miR-141-5p was most differentially expressed. In addition, miR-141-5p was up-regulated in ESA patients’ serum and was negatively correlated with serum β-HCG, P, and E2 levels in ESA patients. Furthermore, the diagnostic value of miR-141-5p in ESA was assessed, finding that the AUC of miR-141-5p was 0.868 (SE = 0.036; 95 % CI = 0.797‒0.939), confirming that miR-141-5p has a diagnostic value for ESA.

However, there are limitations to this study. First, the study elucidated the clinical significance of serum miR-141-5p as a biomarker in ESA, the function, and related molecular mechanisms of miR-141-5p in ESA were not elucidated. It is hoped that the regulatory mechanism of miR-141-5p on ESA can be further explored through *in vitro* cell experiments and *in vivo* animal experiments in future studies. Second, the small number of subjects included may lead to some bias in the experimental results, and a larger study is necessary to verify the results, Furthermore, reports on the combined detection of β-HCG, P, and E2 with miRNAs are required to improve the diagnostic performance of the identified miRNAs.

Serum miR-141-5p is enhanced in ESA patients and is negatively correlated with hormone levels during pregnancy. miR-141-5p has a higher diagnostic value for ESA. The authors expected that the serum miR-141-5p may stand for a more potent indicator to find the signs of ESA at an earlier period, even before the tests based on the ultrasound and β-HCG levels. Collectively, this study would provide instructions for the prevention of ESA and open a new avenue to look for selective surrogates in extrapolating abnormal pregnancies.

## Availability of data and materials

The datasets used and/or analyzed during the present study are available from the corresponding author on reasonable request.

## Ethical approval

All procedures performed in this study involving human participants were in accordance with the ethical standards of the institutional and/or national research committee and with the 1964 Helsinki Declaration and its later amendments or comparable ethical standards. All subjects were approved by the Affiliated Hospital of Guangdong Medical University (n° GD20150721).

## Authors’ contributions

All authors have read and agreed to the published version of the manuscript.

## Funding

Not applicable.

## CRediT authorship contribution statement

**XiaoQun Che:** Conceptualization, Data curation, Writing – original draft, Project administration. **Xiao Wang:** Methodology. **LiLian Wang:** Methodology. **LiHua Xu:** Formal analysis. **Lin Zou:** Formal analysis. **TianZhong Ma:** Investigation. **Bi Chen:** Investigation, Data curation, Writing – review & editing.

## Conflict of interest

The authors declare no conflicts of interest.
